# Effects of R-Phase on Mechanical Responses of a Nickel-Titanium Endodontic Instrument: Structural Characterization and Finite Element Analysis

**DOI:** 10.1155/2016/7617493

**Published:** 2016-05-22

**Authors:** Leandro de Arruda Santos, Pedro Damas Resende, Maria Guiomar de Azevedo Bahia, Vicente Tadeu Lopes Buono

**Affiliations:** ^1^Department of Metallurgical and Materials Engineering, School of Engineering, Universidade Federal de Minas Gerais (UFMG), 31270-901 Belo Horizonte, MG, Brazil; ^2^Department of Restorative Dentistry, Faculty of Dentistry, Universidade Federal de Minas Gerais (UFMG), 31270-901 Belo Horizonte, MG, Brazil

## Abstract

The effects of the presence of the R-phase in a near-equiatomic NiTi alloy on the mechanical responses of an endodontic instrument were studied by using finite element analysis. The input data for the constitutive model in the simulation were obtained by tensile testing of three NiTi wires: superelastic austenite NiTi, austenite + R-phase NiTi, and fully R-phased NiTi. The wires were also characterized by X-ray diffraction and differential scanning calorimetry. A commercially available endodontic instrument was scanned using microcomputed tomography, and the resulting images were used to build the geometrical model. The numerical analyses were performed in ABAQUS using load and boundary conditions based on the ISO 3630-1 specification for the bending and torsion of endodontic instruments. The modeled instrument containing only R-phase demanded the lowest moment to be bent, followed by the one with mixed austenite + R-phase. The superelastic instrument, containing essentially austenite, required the highest bending moment. During bending, the fully R-phased instrument reached the lowest stress values; however, it also experienced the highest angular deflection when subjected to torsion. In summary, this simulation showed that NiTi endodontic instruments containing only R-phase in their microstructure would show higher flexibility without compromising their performance under torsion.

## 1. Introduction

Instruments made of stainless steel or nickel-titanium (NiTi) alloys are commonly used in endodontic therapy. In comparison to stainless steel instruments, those made from NiTi alloys exhibit several advantages, such as higher flexibility, which results in fewer aberrations during the shaping of root canals with complex anatomies, less apical transportation, superior biocompatibility, and high corrosion resistance [1–4]. These advantages are related to a property called superelasticity (SE), which is defined as the ability to recover high deformations after the load removal [5–8].

Despite the advantages exhibited by NiTi endodontic files, using these instruments can result in unexpected failure within the root canal. These failures usually occur for two main reasons: flexural fatigue and torsional overloading [9–11]. Flexural fatigue occurs when the instrument is used to shape curved canals and is subjected to cycles of tensile-compressive stresses because of the rotational movement in the curved region of the canal. Torsional overloading takes place when the instrument has its movement stopped by friction with the canal's wall whilst the shaft continues to rotate. Thus, bending and torsion are the main loading conditions to which the instruments are subjected during use. This makes flexibility and torsional stiffness the two most desirable properties for these instruments.

Most of the efforts devoted to preventing the failure of NiTi endodontic instruments have focused on determining the optimal geometrical characteristics, such as the cross-sectional design, taper, and pitch length [12–19]. Recently, manufacturers have started to invest in new technologies to improve the properties of the superelastic NiTi alloys used for endodontic instruments. One result has been the development of a NiTi wire termed M-Wire (Sportswire LLC, Langley, OK, USA), which is manufactured through a proprietary thermomechanical process. Studies on endodontic rotary instruments fabricated using the M-Wire technology [19–22] indicated that the instruments exhibited significantly increased flexibility and fatigue resistance in comparison to those made of conventional SE NiTi. Alapati et al. [19] reported that M-Wire has higher transformation temperatures and presents an intermediate trigonal phase, named R-phase, coexisting with austenite at room temperature. The proportion of R-phase in M-Wire is a difficult parameter to control and it is not constant. Montalvão et al. [21] associated the presence of R-phase with the higher flexibility and stress relaxation found in M-Wire instruments.

Since R-phase seems to play an important role in the file's behavior, in the present study, we investigated the effects of this phase on the mechanical response of a NiTi endodontic instrument using finite element analysis (FEA). FEA is helpful in assessing information (e.g., stress distribution) that is difficult to obtain experimentally. Three NiTi structures were selected for this study: fully austenitic (conventional superelastic), austenite + R-phase (M-Wire), and fully R-phased. Firstly, the materials were characterized through X-ray diffraction (XRD), differential scanning calorimetry (DSC), and uniaxial tensile tests. Secondly, the data obtained from this characterization were used as inputs to construct finite element models of a commercial instrument. To our knowledge, there is no FEA data regarding a fully R-phased endodontic instrument in the literature.

## 2. Materials and Methods

Wires 1.0 mm in diameter were obtained for each NiTi structure: austenite (conventional superelastic), austenite + R-phase (M-Wire), and R-phase. Dentsply Maillefer (Ballaigues, Switzerland) provided M-Wire wires, while conventional superelastic and R-phase wires were provided by Nitinol Devices & Components Inc. (Fremont, CA, USA).

### 2.1. X-Ray Diffraction

The crystal structure of the materials was characterized by XRD at room temperature. Five segments of each wire were fixed side-by-side in the sample holder of a PANalytical PW1710 diffractometer (PANalytical, Almelo, The Netherlands) with Bragg-Brentano geometry. The specimens were analyzed using Cu-K*α* radiation (*λ* = 0.15418 nm) at 40 kV and 30 mA, using a graphite monochromator. Diffraction spectra were obtained in an angular range of 2*θ* between 20° and 90° at a rate of 0.02°/s. The patterns were indexed using data from the ICSD database [23]. Since several wire segments were used for XRD, the area scanned by the X-ray beam was sufficient to be analyzed by regular XRD. However, Micro-XRD is an interesting alternative to be applied to wire samples, since the analyzed area can be limited to few square micrometers. Some authors already used Micro-XRD in the characterization of orthodontic wires with consistent results [24].

### 2.2. Differential Scanning Calorimetry

Transformation temperatures were determined as the beginning and end of the exothermic/endothermic peaks on the heating and cooling curves recorded by DSC. Three fragments of each wire were cut, weighed, and analyzed with a Shimadzu DSC 60 calorimeter (Shimadzu, Kyoto, Japan) in the temperature range of −80°C to 80°C at a heating and cooling rate of 10°C/min.

### 2.3. Uniaxial Tensile Tests

Wires, 150 mm in length, of each material were tensile tested until rupture in an Instron 5582 testing machine (Instron, Norwood, MA, USA). Three tests for each wire type were performed at room temperature with a strain rate of 1.0 × 10^−3 ^s^−1^, using a clip-on extensometer. In addition, the load/unload behavior was investigated, where samples with the same length described previously were tensile loaded until 4% elongation and then unloaded to zero stress. Material parameters, such as elastic modulus and plateau stresses, were extracted from the stress-strain curves using the BlueHill 2 software.

### 2.4. Finite Element Modeling

**Figure 1:**
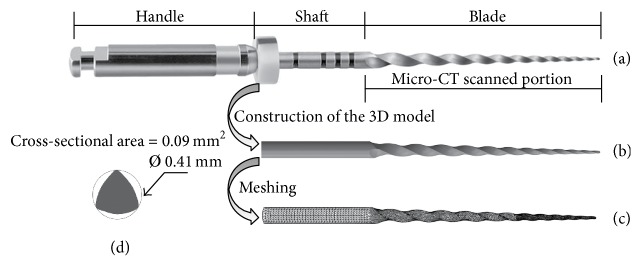
Construction of the finite element model: (a) ProTaper F1 endodontic instrument imaged using micro-CT scanning, (b) its 3D geometrical model, (c) the finite element model, and (d) the cross section of the file at 3 mm from the tip.

The geometry of a commercially available endodontic instrument (ProTaper Universal F1, Dentsply Maillefer, Ballaigues, Switzerland) was used in this study. This instrument can be divided into three main parts (Figure 1(a)): the handle, the shaft, and the blade. The length of the blade is 16.7 mm, with the diameter at the tip of the helix of 0.20 mm and a taper of 7%. The total length (blade and shaft) of the file is 25 mm. In addition, the cross-sectional geometry of the blade is triangular with convex sides. The blade was scanned by microcomputed tomography (micro-CT) (eXplore Locus SP, GE Healthcare, Waukesha, WI, USA) at 12 *μ*m intervals, resulting in 1426 image slices. Micro-CT scanning allowed the capture of the details of the actual geometry of the instrument, eliminating the need for approximations in the geometrical model. The images were further segmented using medical image processing software (Mimics, Materialise HQ, Leuven, Belgium) to build a three-dimensional (3D) model of the blade. The shaft was added at a later stage using SolidWorks 2010 (3D computer-aided design software, Concord, MA, USA). The resultant geometrical model is shown in Figure 1(b). The handle was neglected in the final model for simplification.

**Figure 2:**
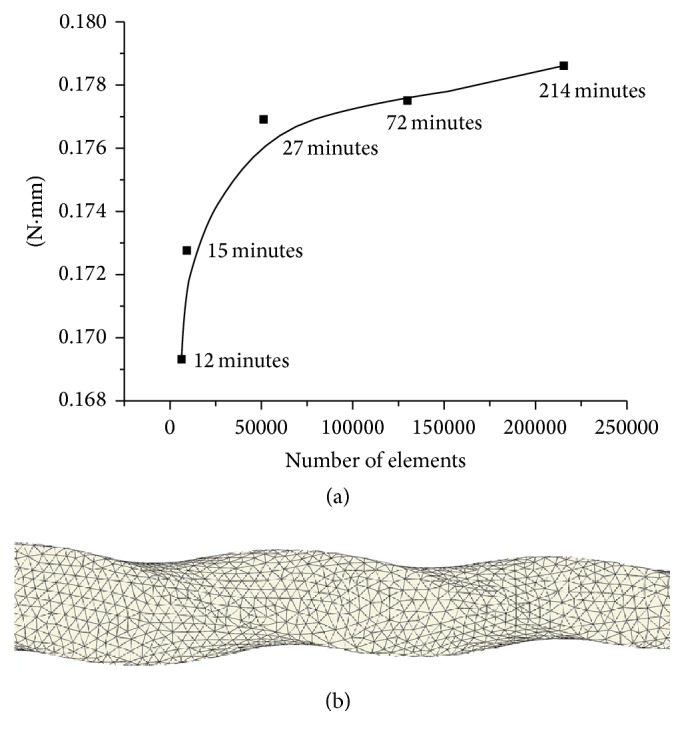
(a) Convergence test: the selected mesh spent 27 minutes to calculate the torque for the chosen condition and (b) a detail of the mesh.

The 3D geometrical model was meshed in ABAQUS 6.9-1 (SIMULIA, Providence, RI, USA) using ten-node quadratic tetrahedral elements (Figure 1(c)). For meshing, the model was divided into three parts in order to change the maximum size of the elements in accordance with the taper of the file. Then, the maximum size of the elements was decreased from the shaft to the tip. A convergence analysis was performed to select the most convenient number of elements in terms of computational time and result accuracy. As convergence tests, the model was subjected to torsion until 5° of displacement and the torque to perform this condition was measured. Taking into account the simulation's time (27 minutes) and the results, the final model consisted of 51,184 elements with 88,344 nodes. The results obtained using different element's size are plotted in Figure 2(a). A detailed view of the selected mesh at 3 mm from the tip of the instrument is also shown (Figure 2(b)).

The necessary parameters to describe the constitutive model for each wire were extracted from the stress-strain curves obtained through the tensile tests performed on the wires. A user-defined subroutine implemented in the ABAQUS code by Auricchio and Petrini [25] was used to describe the behavior of the NiTi alloy.

The mechanical behavior of the endodontic instrument corresponding to different materials was simulated under two load conditions: bending and torsion. The load and boundary conditions used in this study were based on standardized experimental tests in keeping with the ISO 3630-1 specification [26] and can be described as follows:

- Bending: the instrument was held (without translational or rotational movements) at a distance of 3 mm from the tip. The shaft was deflected in one direction until it was at an inclination of 45° to the horizontal. The necessary bending moment to perform this movement was determined for each material.
- Torsion: in this case, the instrument was held at the same place, and a clockwise torsional moment of 3 N·mm was applied at the end of the shaft. This moment value was chosen in terms of time convergence during simulation. The resultant angular displacement was then determined for each material.

**Figure 3:**
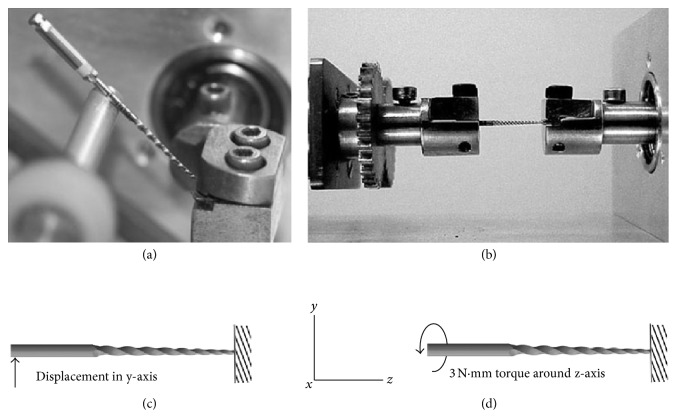
Experimental tests and respective load and boundary conditions: (a) experimental bending test, (b) experimental torsional test, (c) simulated bending test, and (d) simulated torsional test.

Test devices and simulation conditions for both experimental tests are shown in Figure 3. Experimental validation of this methodology for the simulation was performed previously and published by Santos et al. [12].

## 3. Results

### 3.1. Structural Characterization

**Figure 4:**
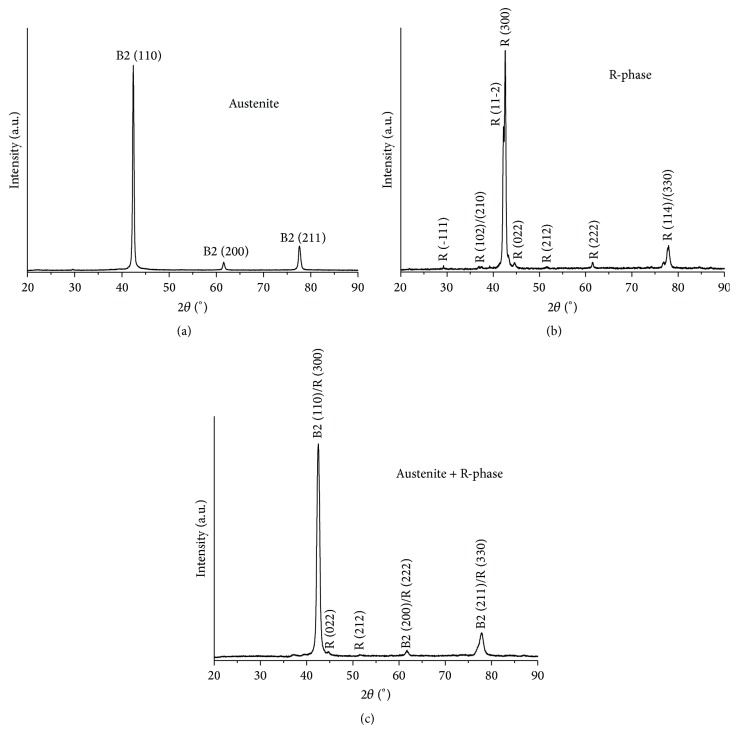
XRD patterns of the three wires used in this study: conventional superelastic (austenite), R-phase, and M-Wire (austenite + R-phase).

Figure 4 shows the XRD patterns of the three wires characterized in this study. The peaks of cubic austenite B2 type (ICSD card #105412) were indexed in the conventional superelastic wire, while peaks of trigonal R-phase (ICSD card #150942) were indexed in the R-phase wire. Through the XRD analysis, it is seen that M-Wire consists of a mixed structure of austenite and R-phase. Nevertheless, the more intense peaks of both phases overlap each other, which makes it difficult to estimate the proportion of phases. According to previous studies [19, 21], R-phase is the minority phase in M-Wire.

### 3.2. Transformation Temperatures

The transformation temperatures determined by DSC are shown in Table 1. The measured austenite start temperature (As) and austenite finish temperature (Af) for conventional superelastic NiTi indicate that above −2°C the alloy is fully austenitic. Only one-step transformation was detected during cooling, which means that this alloy does not form R-phase during the thermal cycle. On the other hand, the analysis of the R-phase wire showed a two-step transformation, as expected. The room temperature was measured to be between the R-phase finish temperature (Rf) and the martensite start temperature (Ms), confirming that this wire is fully R-phased at room temperature. A two-step transformation was also observed in M-Wire. However, the R-phase start and finish temperatures (Rs and Rf, resp.) are quite close to the room temperature, which indicates that the transformation may not be complete at this temperature, resulting in a structure containing both austenite and R-phase. This is consistent with the observations from XRD.

### 3.3. Mechanical Properties

**Figure 5:**
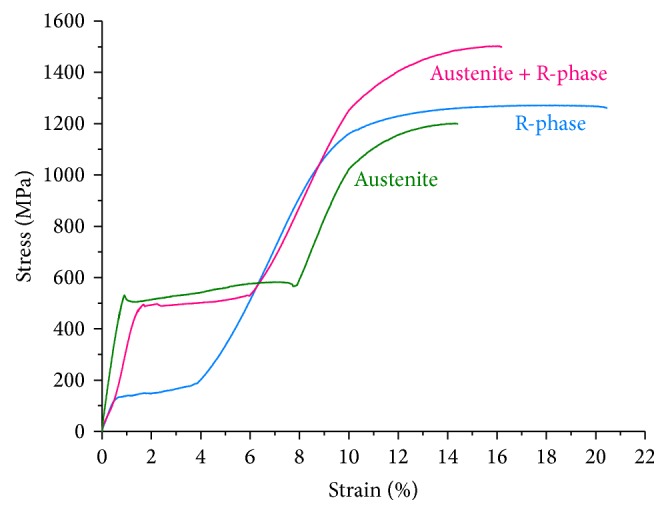
Stress-strain curves for three different NiTi wires used in this study.

Stress-strain curves obtained through tensile tests until rupture are shown in Figure 5. The austenitic wire presents a longer plateau with the highest stress values. R-phase has a quite lower plateau in terms of stress with less elongation. Such differences in terms of mechanical behavior are related to the structure and deformation mechanisms present in these alloys. The plateau regarding the wire with austenite + R-phase was slightly lower than the austenite's plateau but presented a considerable reduction in strain. This may be associated with the presence of R-phase in the structure. Differences in the values of elastic modulus were also found among the wires. The main parameters of the tensile tests are detailed in Table 2 and were used as input data for the constitutive models in the FEA.

**Figure 6:**
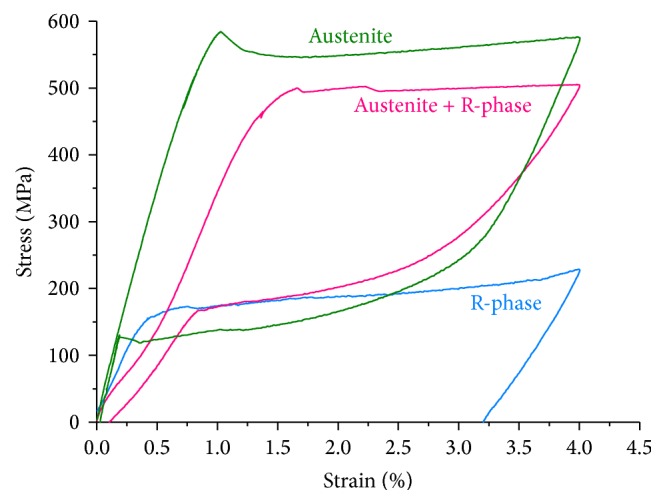
Load/unload behavior for three different NiTi wires used in this study.

The load/unload behavior during uniaxial tensile testing of the NiTi wires is depicted in Figure 6. The austenitic wire showed complete superelastic behavior, with a complete strain recovery after the load removal. It was not observed in the R-phase curve, which shows that, after the load removal, the material recovers only the elastic strain. The R-phase's behavior is also related to the portion of strain not returned in the austenite + R-phase wire. In other words, R-phase presents pseudoplastic behavior.

**Figure 7:**
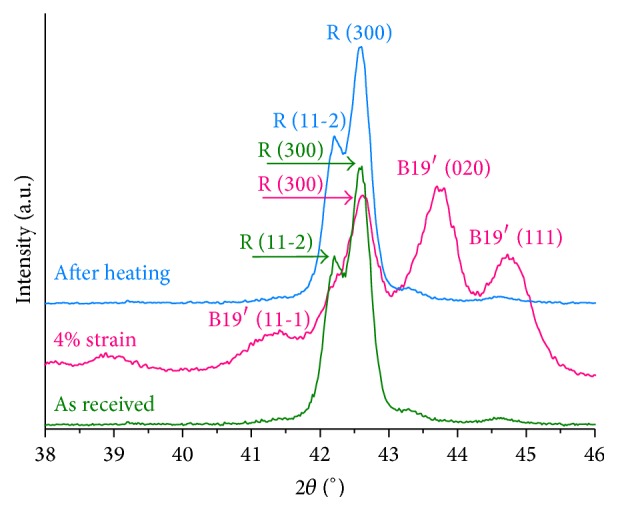
XRD patterns for R-phase as received, after 4% uniaxial strain, and then after heating above Af.

Figure 7 shows the XRD patterns of the same R-phase wire as received, after 4% strain, and then after heating above Af. This is helpful in understanding what happens during the deformation of R-phase and its pseudoplastic cycle. During deformation, B19′ martensite (ICSD card #164156) is stress-induced in the material (pink line in Figure 7), and it is maintained after load removal. Nevertheless, if the material is heated to a temperature above Af (blue line in Figure 7), the atomic structure returns to its initial state, which is only R-phase. Macroscopically, it means that the apparently permanent deformation is recovered, which characterizes pseudoplastic behavior.

### 3.4. Finite Element Modeling

**Figure 8:**
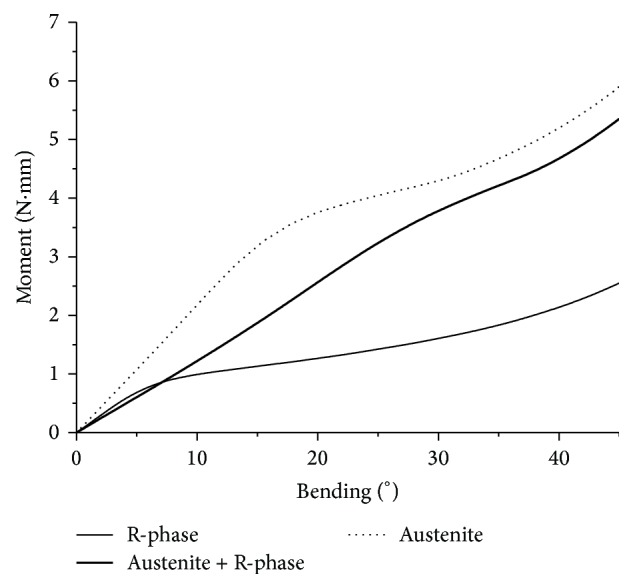
Simulated moment-bending curves for the same endodontic instrument with three different NiTi constitutive models: austenite, austenite + R-phase, and R-phase.

Figure 8 shows the variation in the bending moment caused by the displacement to which the instrument was subjected along the *y*-axis until it was at an inclination of 45° with respect to its long axis. The austenitic NiTi instrument exhibited the highest moment value (5.9 N·mm), followed by the austenite + R-phase instrument (5.3 N·mm) and the R-phase instrument (2.6 N·mm).

**Figure 9:**
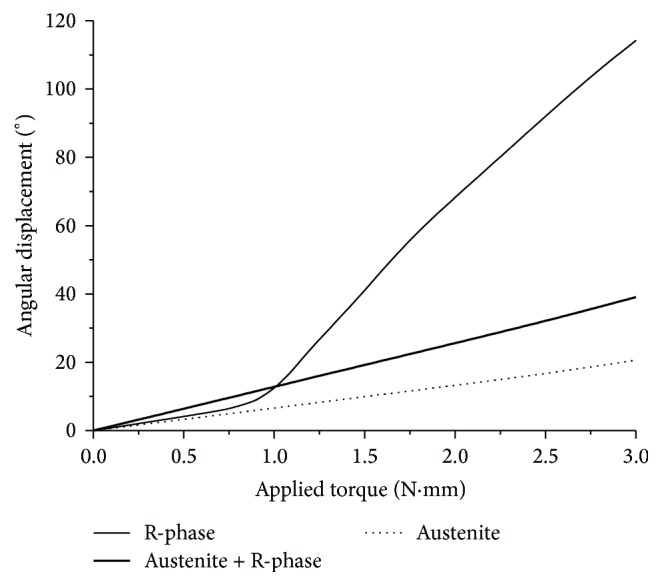
Simulated torsion-torque curves for the same endodontic instrument with three different NiTi constitutive models: austenite, austenite + R-phase, and R-phase.

Figure 9 depicts the angular displacement caused by the torque applied to the instrument. For torques of approximately 1.0 N·mm, the three materials exhibited similar behaviors. However, for greater torques, significant differences were noted, especially in the case of the R-phase instrument, which experienced a large deformation in response to the applied torque. The total angular displacement for the R-phase instrument was 119°. The next highest angular displacement was presented by the austenite + R-phase instrument (39°), followed by the austenitic instrument (22°). These results show that the presence of R-phase in the near-equiatomic NiTi alloy applied to endodontic instruments increases their flexibility and decreases the torsional stiffness markedly.

**Figure 10:**
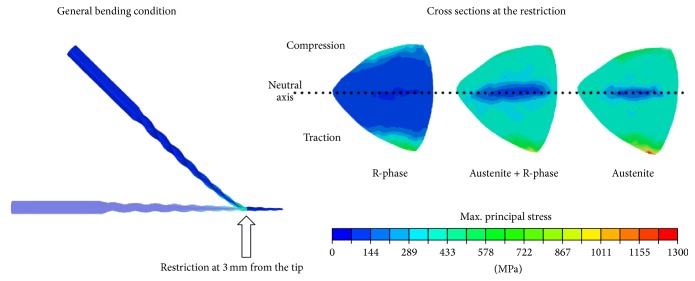
Maximum principal stress distributions in the endodontic instruments subjected to bending.

Figure 10 shows the stress distribution in the cross sections at 3 mm from the tip during the bending simulation. Under this condition, the cross section could be divided into two parts: one that is subjected to tensile strain and another that is under compression, as indicated in Figure 10. Between them, there is a neutral axis where the stress tends to zero. The maximum stress values appear at the periphery of the cross sections close to the restriction position. The calculated maximum stress values were 1,235 MPa for the austenite, 979 MPa for austenite + R-phase, and 801 MPa for R-phase. Thus, the more flexible the instrument, the lower the stress required for bending it at a given angle.

**Figure 11:**
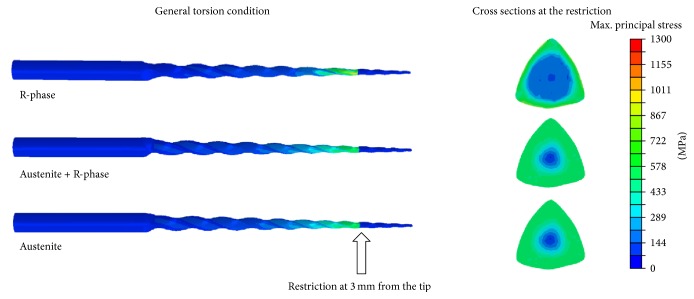
Maximum principal stress distributions in the endodontic instruments subjected to torsion.

The stress distributions along the instruments under torsion are shown in Figure 11. As was the case for the bending condition, the maximum stress values were found at the periphery of the cross sections and were very similar in terms of magnitude: 902 MPa for R-phase, 850 MPa for austenite + R-phase, and 833 MPa for austenite. Although the R-phase instrument exhibited the largest deformation and stress values in response to the applied torque, it had a wider neutral zone, as shown by its cross section in Figure 11. This indicates that most of the deformation and stresses are concentrated only in the periphery and that the core of the instrument is protected from the damaging effects of torsion.

## 4. Discussion

Shape memory alloys (SMA), such as the near-equiatomic NiTi alloy, may present two main properties: SE and shape memory effect (SME). SE, also known as pseudoelasticity, is related to the presence of stable austenite that gives rise to B19′ stress-induced martensite (SIM) when the alloy is subjected to load. Stable austenite means an alloy above Af; thus, the load removal promotes the reverse transformation from SIM to austenite and, consequently, the recovery of large deformations. On the other hand, if this same alloy is cooled down to a temperature below Mf, B19′ martensite will become the stable phase. The strained martensite maintains its deformed state after the load removal. However, heating the strained martensite above Af will again lead to reverse transformation followed by shape recovery. This is called SME, also known as pseudoplasticity [5, 6]. This discussion reveals the importance of determining the transformation temperatures of these alloys, as was conducted in this work through DSC. Table 1 gives different transformation temperatures between cooling and heating paths, which means that the phase transformations follow a thermal hysteresis loop. Besides B19′ martensite and austenite, there is a third phase, called R-phase, that is formed in some cases as an intermediate phase during transformations between austenite and martensite. In most cases, as well as in the present study, R-phase appears only during cooling. However, Brantley et al. [27] also reported the presence of this intermediate phase during heating in a commercially available superelastic NiTi alloy by using temperature-modulated DSC (TMDSC). This technique provides greater resolution than conventional DSC and may reveal details of the transformation, which are not otherwise detected, such as the nonreversing heat-flow component of the transformation.

The transformation temperatures may be shifted in response to thermomechanical processing, which means that it is possible to obtain stable austenite, R-phase, or martensite at room/work temperature depending on the alloy's manufacturing history. It is still possible to obtain an alloy containing a mixture of these phases, which is the case of M-Wire technology. The mechanical properties of the NiTi alloy used to manufacture endodontic instruments are strongly affected by the phases present. Structural changes, introduced by thermomechanical treatments, constitute a modern technique of developing new endodontic instruments with improved mechanical properties [19, 20, 28, 29]. One of the recent efforts is the development of files containing R-phase. Twisted Files (TF; SybronEndo, Orange, CA) is a technology released in the market and claimed by its manufacturers to have a fully R-phased structure. Several works [30–33] evaluated the mechanical behavior of these files and reported improvements in the fatigue resistance of the files in relation to more conventional NiTi rotary files. Nevertheless, none of these studies characterized the alloy structure to confirm the presence of the R-phase. Thus, the role of the R-phase on the mechanical behavior of an endodontic instrument is not completely understood.

The stress-strain curves shown in Figure 5 can be divided into three main regions: (1) linear elastic, (2) plateau, and (3) second linear elastic region. After this, the material undergoes plastic deformation. Under uniaxial tensile load of superelastic NiTi, austenite is elastically deformed (region 1) and then transformed into martensite (region 2); finally, the stress-induced martensite is elastically deformed (region 3). If the plastic regime is not reached, the complete strain is recovered by reverse transformation (Figure 6). Despite the stress and strain values, R-phase presents a similar behavior under loading, with the induction of martensite and consequent plateau. However, the deformation is not returned by unloading, even with R-phase being the more stable structure. The interfaces between R-phase and the martensite variants need more energy to move and promote the reverse transformation during unloading. This is confirmed by Figure 7 that shows the fully reversed R-phase XRD pattern after deformation and heating. The unload behavior of R-phase is also useful in understanding the responses given by M-Wire (Figure 6). Since the amount of residual strain after load removal is related to R-phase, it is reasonable to affirm that this amount is proportional to the quantity of R-phase contained in the alloy. This is consistent with previous observations [19] that R-phase is the minority phase in M-Wire.

In general, when a new NiTi wire is released on the market, it is used to produce files with a particular geometry. The geometry of the file will also influence its performance, and comparing only the effects of different materials becomes a difficult task. FEA is a suitable tool for evaluating the performance of new NiTi wires used to produce endodontic instruments because this method allows the same geometrical model to be used with different constitutive models. Further, through this method, it is even possible to evaluate the behavior of endodontic instruments fabricated with alloys not yet on the market. Another advantage of the FEA method is that it is possible to calculate the stress distribution along the file, which is considerably difficult to do experimentally.

With FEA, it was possible to estimate the effects of R-phase on the instrument's flexibility and observe that, even in small quantities, R-phase contributes favorably in this matter, as in the case of M-Wire. Analyzing the bending curves shown in Figure 8, it is seen that, up until 20° of bending, a significant difference is observed between the moments required to bend the austenitic and the M-Wire instruments. However, after 20°, the difference tends to decrease and the curves become similar. Because M-Wire contains R-phase, the forces required to induce martensite from this phase are lower than those from austenite. The consequence is that the flexibility of M-Wire increases initially. However, after a certain point, at which the fraction of R-phase is fully transformed into martensite, the bending behavior of M-Wire becomes similar to that of austenitic NiTi. Similar findings have been reported by Pereira et al. [20] using bending-displacement curves obtained by means of three-point bending tests performed on NiTi wires. The effect of the stable R-phase on the flexibility is confirmed by the bending-moment relationship calculated for a fully R-phased instrument shown in Figure 8; this instrument exhibited a dramatic increase in flexibility compared to the other two instruments.

In SMA, flexibility is related not only to elastic modulus but also to the stresses developed during the stress-strain plateau, once the consequent strain in this stage is recoverable. R-phase presents the lowest elastic modulus and plateau stress values among the studied NiTi alloys. Clinically, this means an instrument that will easily follow the canal's geometry and prevent further aberrations and apical transportation. Another consequence of using R-phase as the constitutional material for endodontic instruments is the absence of the rebound effect with unloading. After shaping, the instrument would remain deformed, and this would limit file reuse. However, NiTi endodontic instruments undergo autoclave sterilization after use, and this is conducted at temperatures well above their Af. Thus, the original shape of the file is restored by SME. Recently, a new NiTi wire was released on the market under the commercial name of Controlled Memory NiTi (DS Dental, Johnson City, TN, USA); this alloy exhibits behavior similar to that of fully R-phased NiTi, including the ability to return to its original shape after being sterilized in an autoclave [9, 34, 35]. However, this wire is not available on the market, and it is only found in the form of the final product, which makes mechanical tests, such as tensile tests, difficult to perform.

Measuring the magnitudes of the tensile and compressive stresses developed in an endodontic file, once the instrument has been successively rotated during clinical use in curved root canals, is very important. These stresses are alternated in the curvature region, which usually leads to instrument fatigue and failure. The fatigue life varies inversely with the amplitude of the maximum stress to which the NiTi instrument is subjected in the root canal procedure [36]. Thus, even under static simulation conditions, it is possible to estimate which instrument is the most resistant to flexural fatigue from the maximum stress values obtained under bending. Figure 10 shows that the R-phase instrument exhibited low stress values under bending, indicating that R-phase tends to be more fatigue resistant. This is supported by the findings of Figueiredo et al. [37] who studied the fatigue life of martensite. They concluded that martensite has a fatigue life 100 times greater than that of austenite. This behavior is attributable to the crack propagation mechanism in martensite, which occurs through the large number of branched cracks that are formed along the numerous interfaces present in the martensitic structure. The crack propagation is very slow because of the dissipation of energy produced by the branching of the cracks. In superelastic NiTi, the cracks form a limited number of branches; therefore, the energy consumption is lower and the propagation is faster [38]. Martensite is induced more easily in R-phase than in austenite, and it can be favorable for a greater fatigue life.

In general, higher flexibility means lower torsional stiffness, once the file has deformed in response to the applied torque. This can be confirmed by observing the curves in Figure 11, which shows that R-phase exhibits markedly higher angular displacement. However, the stress distributions in the instrument cross sections show that this model conserves its interior structure at low stress levels during torsion, indicating that the large deformation is concentrated almost entirely on the surface. Thus, even under torsion, R-phase instruments may exhibit satisfactory performance against failure due to torsional overloading.

It is important to emphasize that a quasi-static approach was conducted in this study, which is a limitation in the mechanical behavior analysis. Endodontic files are subjected to several strain cycles when at work, and, only by means of very complex numerical modeling, every single aspect of this work could be addressed (e.g., number of cycles, contact forces in the canal). However, to our knowledge, there is still no published study that analyzed the mechanical behavior of endodontic files by FEA taking into account all these variants. Furthermore, as suggestion for future works, TMDSC could be performed on the samples to obtain more precise information about the R-phase transformation, as done by Brantley et al. [27]. Besides that, transmission electron microscopy (TEM) should give complimentary information on the R-phase transformation study.

## 5. Conclusions

The mechanical behavior of a NiTi endodontic file was studied through FEA using three different constitutive models: austenite, austenite + R-phase, and R-phase. The following conclusions can be drawn:

- The R-phase instrument showed the highest flexibility when compared with other instruments. The R-phase demands a lower moment to be applied on an instrument during bending until 45°.
- Under torsion, the R-phase instrument presented the highest angular deflection in response to a torque of 3 N·mm. This means that the presence of R-phase increases the flexibility but decreases the torsional stiffness of the endodontic instrument.
- The results from FEA show that the R-phase instrument presents the lowest stress values under bending, which is expected when a lower moment is required to bend an instrument. Nevertheless, the same material presented stresses only near the surface during the torsional simulation. Thus, aside from the larger deformation imposed on this instrument during torsion, its core is preserved from the damaging effects of this loading condition.
- Although the simulated conditions used in this study were quasi-static, the stress values calculated during the bending tests can indicate a longer fatigue life for instruments made of R-phase NiTi, since the fatigue mechanisms are related directly to the stress levels reached during the flexural work of the instruments.

## Figures and Tables

**Table 1:** Transformation temperatures measured by DSC.

Heat flow	Transformation temperatures (°C)	Austenite	Austenite + R-phase	R-phase
Heating	As	−25	28	40
	Af	−2	47	51

Cooling	Rs	—	26	39
	Rf	—	24	34
	Ms	−5	19	−13
	Mf	−22	15	−39

**Table 2:** Parameters obtained through tensile tests and used to describe the constitutive models for simulation.

Parameter (units)	Description	Values		
		Austenite	Austenite + R-phase	R-phase
*E*_BP_ (MPa)	Elastic modulus before plateau	54,390	38,680	25,210
*ν*	Poisson's ratio	0.33	0.33	0.33
*E*_AP_ (MPa)	Elastic modulus after plateau	22,510	18,970	19,700
*ε* (%)	Plateau's strain	7	4.2	3.2
*σ*_PS_ (MPa)	Stress at the plateau's start	530	488	134
*σ*_PE_ (MPa)	Stress at the plateau's end	570	528	187
*T*_0_ (°C)	Reference temperature	25	25	25
*σ*_*M*_ (MPa)	Maximum stress before plasticity	983	1,285	1,062
